# Potentially Functional Variants of *PLCE1* Identified by GWASs Contribute to Gastric Adenocarcinoma Susceptibility in an Eastern Chinese Population

**DOI:** 10.1371/journal.pone.0031932

**Published:** 2012-03-06

**Authors:** Mengyun Wang, Ruoxin Zhang, Jing He, Lixin Qiu, Jin Li, Yanong Wang, Menghong Sun, Yajun Yang, Jiucun Wang, Jingmin Yang, Ji Qian, Li Jin, Hongxia Ma, Qingyi Wei, Xiaoyan Zhou

**Affiliations:** 1 Cancer Research Laboratory, Fudan University Shanghai Cancer Center, Shanghai, China; 2 Department of Medical Oncology, Fudan University Shanghai Cancer Center, Shanghai, China; 3 Department of Abdominal Surgery, Fudan University Shanghai Cancer Center, Shanghai, China; 4 Department of Pathology, Fudan University Shanghai Cancer Center, Shanghai, China; 5 Ministry of Education Key Laboratory of Contemporary Anthropology, State Key Laboratory of Genetic Engineering, School of Life Sciences, Fudan University, Shanghai, China; 6 Fudan-Taizhou Institute of Health Sciences, Taizhou, Jiangsu, China; 7 Department of Epidemiology and Statistics, School of Public Health, Nanjing Medical University, Nanjing, Jiangsu, China; 8 Department of Epidemiology, The University of Texas MD Anderson Cancer Center, Houston, Texas, United States of America; 9 Department of Oncology, Shanghai Medical College, Fudan University, Shanghai, China; Innsbruck Medical University, Austria

## Abstract

**Background:**

Recent genome-wide association studies (GWAS) have found a single nucleotide polymorphism (SNP, rs2274223 A>G) in *PLCE1* to be associated with risk of gastric adenocarcinoma. In the present study, we validated this finding and also explored the risk associated with another unreported potentially functional SNP (rs11187870 G>C) of *PLCE1* in a hospital-based case-control study of 1059 patients with pathologically confirmed gastric adenocarcinoma and 1240 frequency-matched healthy controls.

**Methodology/Principal Findings:**

We determined genotypes of these two SNPs by the Taqman assay and used logistic regression models to estimate odds ratios (ORs) and 95% confidence intervals (95% CI). We found that a significant higher gastric adenocarcinoma risk was associated with rs2274223 variant G allele (adjusted OR = 1.35, 95% CI = 1.14–1.60 for AG+GG vs. AA) and rs11187870 variant C allele (adjusted OR = 1.26, 95% CI = 1.05–1.50 for CG+CC vs. GG). We also found that the number of combined risk alleles (i.e., rs2274223G and rs11187870C) was associated with risk of gastric adenocarcinoma in an allele-dose effect manner (*P*_trend_ = 0.0002). Stratification analysis indicated that the combined effect of rs2274223G and rs11187870C variant alleles was more evident in subgroups of males, non-smokers, non-drinkers and patients with gastric cardia adenocarcinoma. Further real-time PCR results showed that expression levels of *PLCE1* mRNA were significantly lower in tumors than in adjacent noncancerous tissues (0.019±0.002 vs. 0.008±0.001, *P*<0.05).

**Conclusions/Significances:**

Our results further confirmed that genetic variations in *PLCE1* may contribute to gastric adenocarcinoma risk in an eastern Chinese population.

## Introduction

Gastric cancer is one of the leading causes of cancer-related deaths in the world, accounting for 8% of the total new cases and 10% of total deaths worldwide in 2008 [1]. The high incidence occurs particularly in areas of East Asia (especially China and Japan), Eastern Europe and parts of Central and South America [2]. In China, gastric cancer remains to be a major cancer burden, and two thirds of the incident cases take place in rural areas, ranking the third most common cancer in China [3]. Although the mortality rate has declined due to improvement in social-economic environment, lifestyle, nutrition intake and health care system, there are still urgent needs for early diagnose and cancer prevention because of poor prognosis and lack of novel treatments for this disease.

Gene-environment interaction continues to be an acknowledged cause for gastric cancer carcinogenesis [4]. Previously established environmental factors associated with risk of gastric cancer include *Helicobacter pylori* infection [5], dietary habits (e.g., high intake of salt-preserved and nitrated foods), smoking, pernicious anemia and a history of partial gastrectomy [6]. Extensive epidemiological studies have demonstrated that genetic variants, particularly single nucleotide polymorphisms (SNPs), are likely to modulate the effect of environmental risk factors through modifying functions of various biological pathways involved in gastric carcinogenesis in response to environmental exposure.

Previous molecular epidemiologic studies on associations between SNPs and risk of gastric cancer primarily focused on those involving inflammatory cytokines (like IL-1β and TNF-α) [7], metabolic enzymes (such as the cytochrome P450 superfamily [8], glutathione S-transferase family [9], folate metabolism related enzymes [10]) and DNA repair genes [11], [12]. Recently, two genome-wide association studies (GWASs) [13], [14] have reported a shared susceptible locus at 10q23 (rs2274223A>G, exon 26) in the *PLCE1* gene associated with risk of gastric adenocarcinoma. In one GWAS, *PLCE1* rs2274223 was reported to be associated with risk of both esophageal squamous cell carcinoma and gastric cardia adenocarcinoma in northern Chinese populations [13], and the other GWAS reported a similar finding in ethnic Chinese populations in other geographic areas [14], in which an association was found only in gastric cardia adenocarcinoma but not gastric noncardia adenocarcinoma. Such a finding was replicated in another independent case-control study of gastric cancer in a Chinese population [15].

The *PLCE1* gene encodes phospholipase C [16] that catalyzes the hydrolysis of polyphatidylinositol 4,5-bisphosphate into second messengers such as inositol 1,4,5-trisphosphate and 4,5-diacylglycerol [17], [18]. The phospholipase C also functions as an effector protein in Ras-, Rho- and G_αβγ_-mediated signaling [19], a mediator of extracellular signaling, further affecting cell motility, fertilization and sensory transduction [20]. Therefore, we hypothesized that SNPs in the *PLCE1* gene are associated with gastric cancer risk in eastern Chinese populations. To test this hypothesis, in addition to the reported rs2274223A>G, we also selected another potentially functional SNP rs11187870G>C in the 3′UTR miRNA binding site of *PLCE1* identified by HapMap and SNPinfo (http://snpinfo.niehs.nih.gov/), which is in incomplete linkage disquilibrium (LD) with rs2274223 (*r^2^* = 0.65, **D' = 0.92**) as well as in high LD with three of the novel SNPs revealed in GWAS of gastric cancer (rs753724 G>T, *r^2^* = 0.823; rs11187842 C>T, *r^2^* = 0.823; rs3781264 T>C, *r^2^* = 0.823) [14]. We further measured the *PLCE1* mRNA expressions in paired tissue samples of different genotypes to investigate the possibly functional role of variant rs2274223 in the etiology of gastric adenocarcinoma.

## Materials and Methods

### Study Population

The study population consisted of 1,059 Han Chinese patients with newly diagnosed and histopathologically confirmed gastric cancer from Fudan University Shanghai Cancer Center (Shanghai, China) between 2009 and 2010. All patients came from the Eastern China, including Shanghai, Jiangsu and the surrounding regions. Exclusion criteria included gastric adenosquamous carcinoma, squamous cell carcinoma, neuroendocrine tumor, stromal tumor, metastasized cancer from other organs and esophageal tumors. In addition, 1,240 cancer-free controls were recruited from Taizhou Longitudinal Study (TZL) at the same period with the selection criteria including no individual history of cancer [21]. These cancer-free Han Chinese controls were frequency matched to the cases on age (±5 years) and sex. At recruitment, each participant was personally interviewed to gather demographic data (such as age, sex, and ethnicity) and environmental exposure history, including smoking and alcohol consumption. After interview, each participant donated approximately 10 mL of blood, of which 1 mL was used for genomic DNA extraction. This study was approved by the Institutional Review Board of Fudan University Shanghai Cancer Center.

### SNP Selection and Genotyping

*PLCE1* is located on chromosome 10q23 with 32 exons (NM_001165979.1), encoding for a protein of 2286 amino acids (BC144286.1) (NCBI dbSNP database: http://www.ncbi.nlm.nih.gov/). Besides the non-synonymous rs2274223 SNP identified in the published GWAS [13], [14], we searched the NCBI dbSNP database for other common, potentially functional SNPs of *PLCE1* by using a set of tools at the website SNPinfo (http://snpinfo.niehs.nih.gov/). Potentially functional SNPs were defined as non-synonymous SNPs with minor allele frequency (MAF) >5%, SNPs located at the 5′ UTR, 3′UTR and splice sites. The rs11187870 SNP located in the miRNA binding site of the 3′UTR and rs3203713 of the 5′ UTR were selected by SNPinfo, but rs3203713 was not detected in Asians as listed in the NCBI dbSNP database, nor in our study population (data not shown). As a result, we genotyped two SNPs (rs2274223A>G and rs11187870G>C) of *PLCE1* using genomic DNA isolated from blood samples using the QIAamp DNA blood maxi kit (Qiagen, Valencia, CA). Genotyping for SNPs rs2274223 and rs11187870 was performed using the Taqman assays (Applied Biosystems, Foster City, CA) with a 7900 HT sequence detector system (Applied Biosystems). As recommended by the company, four negative controls (without DNA template) and two duplicated samples were included in each 384-plate for the quality control. The assays were repeated for 5% of the samples, and the results were 100% concordant.

### Tissue Preparation

According to genotyping results of rs2274223, we further performed the real-time PCR analysis by using surgically removed tissues from 48 patients with different genotypes (including 17 AA, 15 AG and 16 GG genotype carriers). All of these patients had undergone gastrectomy for gastric adenocarcinoma without preoperative treatment and provided written informed consent. Gastric adenocarcinoma tumor and adjacent normal tissues were dissected and evaluated by a pathologist after surgery, transferred into liquid nitrogen immediately after resection and stored at −80°C until use at Department of Pathology and Tissue Bank of Shanghai Cancer Center, Fudan University. This use of these specimens was approved by the institutional review board.

### Real-Time PCR

RNA was extracted from samples of tumor and corresponding normal tissues with the TRIzol reagent (Invitrogen) according to the manufacturer's instructions. One micrograms of each RNA sample was subjected to reverse transcription (RT) with the PrimeScript® RT Master Mix system, according to the manufacturer's instructions (TAKARA). The primers used for the real-time RT-PCR were: 5′-CCTGGGCATAAGCACTACCAAG-3′ and 5′-GTCTTGAGGATCAGAACCACTCC-3′ for *PLCE1*; 5′-AGCCTCGCCTTTGCCGAT-3′ and 5′ CTTCTGACCCATGCCCACC 3′ for β-actin. A total of 1 µl of the resulting cDNA reaction mixture was used to set up the real-time PCR, using the ABI Prism 7900 HT Sequence Detection System with the following cycling conditions: (i) 30 s at 95°C and (ii) 40 cycles, with every cycle consisting of 5 s at 95°C, 30 s at 60°C. Each sample was run in triplicate, and β-actin was used as an internal reference under the same experimental conditions. The PCR products were analyzed by melting curves and agarose gel electrophoresis to monitor specificity. The values were obtained through normalizing *PLCE1* copies to β-actin copies. For each sample, the difference in threshold cycles for each *PLCE1* copy was calculated by 2-ΔCT (ΔCT = Avg. PLCE1 CT - Avg.β-actin CT). 2-ΔΔCT was also calculated and ΔΔCT was determined as follows: ΔΔCT = ΔCT_tumor_-ΔCT_normal_, whereΔCT_tumor_ = Ct_tumor/PLCE1_-Ct_tumor/β-actin_ and ΔCT_normal_ = Ct_normal/PLCE1_-Ct_normal/β-actin_.

### Statistical Analysis

Pearson's χ^2^ test was used to evaluate differences in the distributions of categorical variables, including selected demographic variables, the known risk factors, such as smoking and drinking status, as well as frequencies of *PLCE1* genotypes between the cases and controls. The Hardy–Weinberg equilibrium of the control genotype distributions was tested by a goodness-of-fit χ^2^ test. Both univariate and multivariate logistic regression analyses were conducted to estimate odds ratios (ORs) and 95% confidence intervals (CIs) for associations between different genotypes and risk of gastric cancer and were stratified by age, sex, smoking/drinking status and primary tumor site. The homogeneity tests were also performed to test for any difference in the risk estimates between the strata. Haplotype frequencies and individual haplotypes were estimated and analyzed using Statistical Analysis Software PROC HAPLOTYPE. Paired-Sample Student's *t* test was applied for the comparison of mRNA levels between paired samples of different genotype. All tests were two-sided using the Statistical Analysis Software (v.9.1 SAS Institute, Cary, NC), and *P*<0.05 was considered statistical significant.

## Results

### Characteristics of the Study Population

As shown in **Table 1**, the final analysis included 1,059 cases and 1,240 controls who were adequately frequency matched by age and sex (*P* = 0.425 and 0.117, respectively). The mean age was almost the same between cases (58.40±11.32 years) and controls (58.40±11.99 years) The cases had more smokers and drinkers (38.1% and 23.7%, respectively), compared with the controls (34.0% and 18.1%, respectively; *P* = 0.041 and *P* = 0.001, respectively). In addition, more cases reported to be heavy smokers than did the controls (21.72% vs. 17.34% for <22.5 pack-years; *P* = 0.028). These variables (i.e., age, sex, smoking status and drinking status) were further adjusted for in later multivariate logistic regression analyses. Of the cases, 284 (26.82%) had cardia gastric cancers, and 775 (73.18%) had non-cardia gastric cancers.

**Table 1:** Distributions of selected variables in GA cases and cancer-free controls.

Variables	Cases No. (%)	Controls No. (%)	*P* a
All subjects	1,059(100)	1,240 (100)	
Age, yr (Mean±SD)^b^	58.40±11.32	58.40±11.99	
≤58 (median)	507 (47.9)	573 (46.2)	0.425
>58 (median)	552 (52.1)	667 (53.8)	
Sex			0.117
Males	752 (71.0)	843 (68.0)	
Females	307 (29.0)	397 (32.0)	
Smoking status			0.041
Yes	403 (38.1)	421 (34.0)	
No	656 (61.9)	819 (66.0)	
Drinking status			0.001
Yes	251(23.7)	225 (18.1)	
No	808 (76.3)	1015 (81.9)	
Pack-years			0.028
0	656 (61.95)	819 (66.05)	
≤22.5 (mean)	230 (21.72)	215 (17.34)	
>22.5 (mean)	173(16.33)	206 (16.61)	
Tumor site			
Cardia	284(26.82)		
Non-cardia	775(73.18)		

GA, gastric adenocarcinoma.

a Two-sided *χ^2^* test for distributions between cases and controls.

b Data are mean ± SD and P value from Student's *t* test.

### Associations between PLCE1 Genotypes and Risk of Gastric Cancer

Genotype distributions of the two selected SNPs (rs2274223 and rs11187870) in cases and controls are summarized in **Table 2**. The observed genotype frequencies for the two SNPs agreed with the expected from the Hardy–Weinberg equilibrium in the controls (*P* = 0.224 for rs2274223 and *P* = 0.688 for rs11187870). The genotype distributions between the cases and controls were significantly different for both rs2274223 and rs11187870 (*P* = 0.0012 and *P* = 0.010, respectively). In the dominant model, a significantly increased risk of gastric cancer was associated with variant genotypes (AG+GG) of rs2274223 with an adjusted OR of 1.35 (95% CI = 1.14–1.60) and (CG+CC) of rs11187870 with an adjusted OR of 1.26 (95% CI = 1.05–1.50), compared with the wild-type homozygous genotypes, respectively. In addition, the variant rs2274223 G and rs11187870 C alleles were significantly associated with increased risk of gastric cancer (adjusted OR = 1.26, 95% CI = 1.10–1.45 for rs2274223 G allele; adjusted OR = 1.22, 95% CI = 1.05–1.42 for rs11187870 C allele, respectively), with P values of 0.001 and 0.01, respectively.

**Table 2:** Logistic regression analysis of associations between the genotypes of *PLCE1* and GA risk.

Variants	Genotypes	Cases(N = 1,059)	Controls(N = 1,240)	Crude OR(95% CI)	*P* a	Adjusted OR(95%CI)^b^	*P* b
*PLCE1* rs2274223							
	AA	600 (56.66)	791(63.79)	1.00		1.00	
	AG	399 (37.68)	390 (31.45)	1.35 (1.13–1.61)	**0.0008**	**1.35 (1.13–1.61)**	**0.0008**
	GG	60 (5.66)	59 (4.76)	1.34 (0.92–1.95)	0.125	1.34 (0.92–1.96)	0.123
	AG+GG	459 (43.34)	449 (36.2)	1.35 (1.14–1.59)	**0.0005** d	**1.35 (1.14–1.60)**	**0.0005** d
Allele	A	1,599 (75.5)	1,972 (79.5)	1.00		1.00	
	G	519 (24.5)	508 (20.5)	1.26 (1.10–1.45)	**0.001** f	**1.26 (1.10–1.45)**	**0.001** f
*PLCE1* rs11187870							
	GG	691 (65.25)	870 (70.2)	1.00		1.00	
	CG	329 (31.07)	335 (27.0)	1.24 (1.03–1.48)	**0.022**	**1.24 (1.03–1.49)**	**0.021**
	CC	39 (3.68)	35 (2.8)	1.40 (0.88–2.24)	0.156	1.41(0.88–2.25)	0.150
	CG+CC	368 (34.75)	370 (29.8)	1.25 (1.05–1.49)	**0.012** d	**1.26 (1.05–1.50)**	**0.011** d
Allele	G	1,711 (80.8)	2,075 (83.7)	1.00		1.00	
	C	407 (19.2)	405 (16.3)	1.22 (1.05–1.42)	**0.01** f	**1.22 (1.05–1.42)**	**0.01** f
Combined effect of risk alleles							
	0	579 (54.67)	772 (62.3)	1.00	0.0011^c^	1.00	
	1–2	421 (39.75)	413 (33.3)	**1.36 (1.14–1.62)**	**0.0005**	**1.36 (1.14–1.62)**	**0.0005**
	3–4	59 (5.57)	55 (4.4)	1.43 (0.97–2.09)	0.07	1.43 (0.97–2.09)	0.07
				*P*_trend_ = 0.0002		***P*** **_trend_** b ** = 0.0002**	
	0	579 (54.67)	772 (62.3)	1.00		1.00	
	≥1	480 (45.33)	468 (37.7)	1.37 (1.16–1.62)	**0.0002**	**1.37 (1.16–1.62)**	**0.0002**

GA, gastric adenocarcinoma; SNP, single-nucleotide polymorphism; CI, confidence interval; OR, odds ratio.

a Chi square test for genotype distributions between cases and controls.

b Adjusted for age, sex, smoking status and drinking status in logistic regress models.

c for additive genetic models.

d for dominant genetic models.

f at allelic levels.

The results were in bold, if the 95% CI excluded 1 or *P*<0.05.

**Figure 1:**
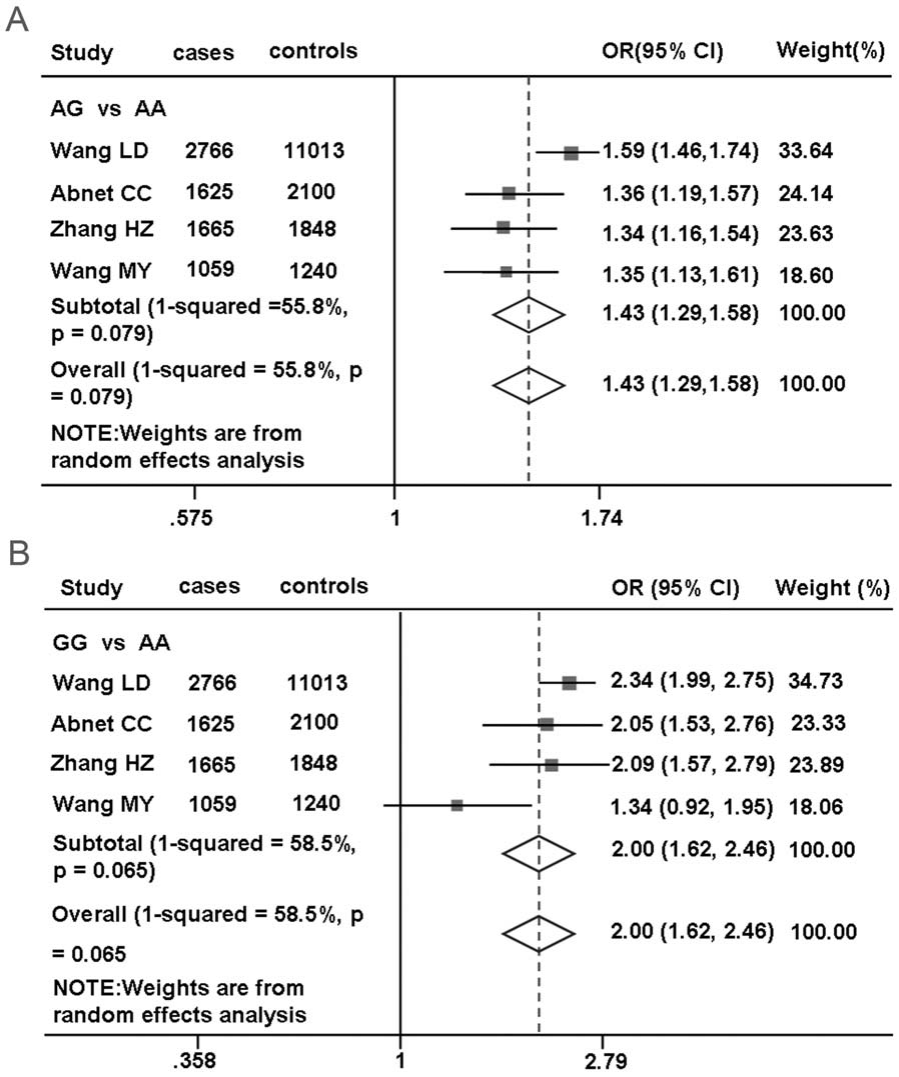
Forest plot showing associations between *PLCE1* rs2274223 and gastric cancer risk. A) The ORs and 95% CIs were obtained using AG vs. AA. B) The ORs and 95% CIs were obtained using GG vs. AA. The axis corresponds to the OR. The diamonds and the horizontal bars represent the overall ORs with 95% CIs given by their width.

To explore the independence of rs2274223 and rs11187870 with gastric cancer risk, they were simultaneously included in a logistic regression model with adjustment for other covariates. The strength of the association for rs2274223 (OR = 1.39, 95% CI = 1.07–1.81) was almost unchanged, while the association for rs11187870 (OR = 0.96, 95% CI = 0.73–1.26) was attenuated, indicating that only rs2274223 was independently associated with gastric cancer risk. Finally, we also performed a mini-meta analysis of *PLCE1* rs2274223 with our and the published three other studies (**Figure 1**). Consistently, we found that *PLCE1* rs2274223 variant G allele was significantly associated with an increased risk of gastric cancer (the pooled OR = 1.43; 95% CI = 1.29–1.58 for the AG genotype and OR = 2.00; 95% CI = 1.62–2.46 for the GG genotype, compared with the AA genotype) based on 7115 cases and 16201 controls in our pooled analysis.

We then categorized the putative risk alleles of the two SNPs into the number of combined variant alleles (i.e., rs2274223G and rs11187870C) to further analyze their possible joint effect and potential locus-locus interaction of *PLCE1* SNPs on risk of gastric cancer. As shown in **Table 2**, when we trichotomized the subjects into three groups according to the number of combined variant alleles, the number of observed risk alleles was associated with gastric cancer risk in an allele-dose response manner (*P* for trend = 0.0002). Specifically, when the “0” risk allele group was used as the reference, the increased risk of gastric cancer was 1.36 fold (95% CI = 1.14–1.62) for those who carried “1–2” risk alleles and the risk increased to 1.43 fold (95% CI = 0.97–2.09) for those who carried “3–4” risk alleles. Because relatively fewer subjects carried “3–4” risk alleles, we merged “1–2” and “3–4” into one group of “≥1” risk alleles. As a result, individuals who carried 1–4 risk alleles exhibited a significantly increased risk of gastric cancer (adjusted OR = 1.37, 95% CI = 1.16–1.62), compared with those who did not carry any risk alleles. Then, we used this combined group for further stratified analysis.

### Stratification Analysis

As shown in **Table 3**, the stratification analysis indicated that the risk associated with the combined rs2274223 variant AG/GG genotypes and rs11187870 variant CG/CC genotypes was more evident in males (adjusted OR = 1.37, 95% CI = 1.12–1.67 for rs2274223 AG/GG genotypes; adjusted OR = 1.33, 95% CI = 1.08–1.65 for rs11187870 CG/CC genotypes, respectively), non-smokers (adjusted OR = 1.45, 95% CI = 1.17–1.79 for rs2274223 AG/GG genotypes; adjusted OR = 1.30, 95% CI = 1.04–1.62 for rs11187870 CG/CC genotypes, respectively), non-drinkers (adjusted OR = 1.38, 95% CI = 1.14–1.66 for rs2274223 AG/GG genotypes; adjusted OR = 1.22, 95% CI = 1.00–1.48 for rs11187870 CG/CC genotypes, respectively) and subjects with cardia cancer (adjusted OR = 2.07, 95% CI = 1.59–2.70 for rs2274223 AG/GG genotypes; adjusted OR = 1.86, 95% CI = 1.42–2.43 for rs11187870 CG/CC genotypes) compared with those without any variant genotypes. Consistently, the increased gastric cancer risk associated with any (≥1) of the variant risk alleles or genotypes was also more pronounced among non-smokers (adjusted OR = 1.51, 95% CI = 1.22–1.86) and non-drinkers (adjusted OR = 1.40, 95% CI = 1.16–1.69), compared with those without any (0) variant risk alleles. Additional analysis with groups of pack-years smoked did not generate more striking data (data not shown) than did the smoking status. Further homogeneity tests suggested that there were no differences in the risk estimates between these strata except for tumor site. There was no statistical evidence for interactions between the variant genotypes and any of the tested variables (i.e., age, sex, smoking status and alcohol consumption) on risk of gastric cancer (data not shown).

**Table 3:** Stratification analysis for associations between *PLCE1* variant genotypes and GA risk.

Variables	rs2274223(cases/controls)		Adjusted OR^a^ (95% CI)	*P* a	rs11187870(cases/controls)		Adjusted OR^a^ (95% CI)	*P* a	Combined effect of risk alleles(cases/controls)		Adjusted OR^a^ (95% CI)	*P* a
	AA	AG/GG			GG	CG/CC			0	≥1		
Age, yr												
≤58 (median)	302/380	205/193	**1.33 (1.03–1.70)**	**0.027**	349/413	158/160	1.16 (0.88–1.49)	0.312	293/370	214/203	**1.32 (1.03–1.69)**	**0.028**
>58 (median)	298/411	254/256	**1.37 (1.09–1.73)**	**0.007**	342/457	210/210	**1.35 (1.06–1.71)**	**0.014**	286/402	266/265	**1.42 (1.13–1.78)**	**0.003**
Gender												
Males	417/530	335/313	**1.37 (1.12–1.67)**	**0.002**	482/593	270/250	**1.33 (1.08–1.65)**	**0.007**	401/516	351/327	**1.39 (1.14–1.69)**	**0.001**
Females	183/261	124/136	1.33 (0.97–1.82)	0.075	209/277	98/120	1.08 (0.78–1.50)	0.652	178/256	129/141	1.35 (0.99–1.84)	0.062
Smoking status												
Never	362/526	294/293	**1.45 (1.17–1.79)**	**<0.001**	423/576	233/243	**1.30 (1.04–1.62)**	**0.020**	348/517	308/302	**1.51 (1.22–1.86)**	**<0.001**
Ever	238/265	165/156	1.18 (0.88–1.56)	0.271	268/294	135/127	1.17 (0.87–1.58)	0.300	231/255	172/166	1.15 (0.86–1.52)	0.349
Drinking status												
Never	462/651	358/364	**1.38 (1.14–1.66)**	**<0.001**	536/708	284/307	**1.22 (1.00–1.48)**	**0.049**	446/636	374/379	**1.40 (1.16–1.69)**	**<0.001**
Ever	138/140	101/85	1.27 (0.86–1.87)	0.225	155/162	84/63	1.46 (0.97–2.21)	0.069	133/136	106/89	1.29 (0.88–1.89)	0.194
Tumor site												
Cardia	129/791	155/449	**2.07(1.59–2.70)**	**<0.001**	158/870	126/370	**1.86(1.42–2.43)**	**<0.001**	124/772	160/468	**2.08(1.59–2.7)**	**<0.001**
Non-cardia	479/791	296/449	1.10(0.92–1.33)	0.30	549/870	226/370	0.98(0.81–1.20)	0.87	467/772	308/468	1.10(0.92–1.33)	0.03

GA, gastric adenocarcinoma; SNP, single-nucleotide polymorphism; CI, confidence interval; OR, odds ratio.

a Obtained in logistic regression models with adjustment for age, sex, smoking status and drinking status.

The results were in bold, if the 95% CI excluded 1 or *P*<0.05.

### Haplotype and Stratification Analyses

By using the SAS PROC HAPLOTYPE program, we estimated four possible hapolotypes from the observed genotypes of the two SNPs and assessed their associations with gastric cancer risk (**Table 4**). When the most common haplotype A_rs2274223_G_rs11187870_ was used as the reference, G_rs2274223_C_rs11187870_ and G_rs2274223_G_rs11187870_ haplotypes were both associated with a significantly increased risk of gastric cancer (adjusted OR = 1.24, 95% CI = 1.06–1.45, adjusted OR = 1.34, 95% CI = 1.04–1.72, respectively), whereas subjects who carried the A_rs2274223_C_rs11187870_ haplotype had non-significantly increased risk of gastric cancer (adjusted OR = 1.33, 95% CI = 0,74–2.38), which may be due to the limited sample size of this subgroup.

**Table 4:** Haplotype analysis for genotypes of *PLCE1* and GC risk.

*PLCE1* haplotypes^a^	Haplotype frequencies				Crude OR(95% CI)	Adjusted OR^a^(95% CI)	*P* a
	Cases(N = 2,118)		Controls(N = 2,480)				
	N	%	N	%			
A_rs2274223_G_rs11187870_	1575	74.4	1950	78.6	1.00	1.00	
G_rs2274223_C_rs11187870_	383	18.1	383	15.4	**1.24 (1.06–1.45)**	**1.24 (1.06–1.45)**	**0.007**
G_rs2274223_G_rs11187870_	136	6.4	125	5.0	**1.35 (1.05–1.73)**	**1.34 (1.04–1.72)**	**0.024**
A_rs2274223_C_rs11187870_	24	1.1	22	0.9	1.35 (0.75–2.42)	1.33 (0.74–2.38)	0.344

a Obtained in logistic regression models with adjustment for age, sex, smoking status and drinking status.

The results were in bold, if the *P* value was significant.

### Real-Time PCR Analysis

**Figure 2:**
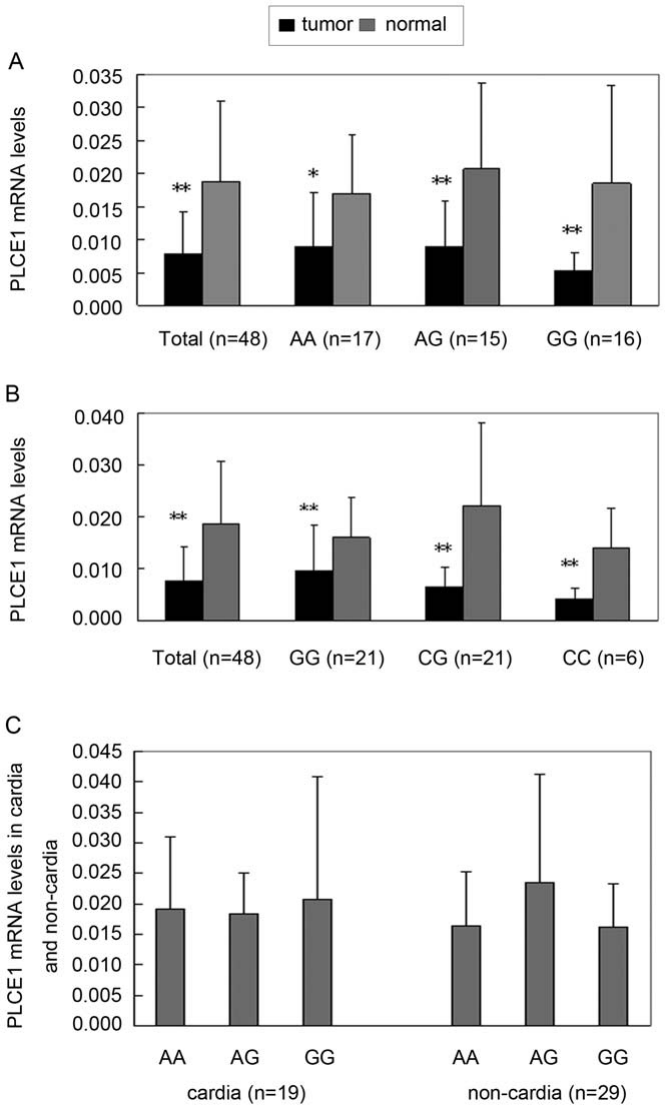
Real-time PCR quantification of expression of *PLCE1* mRNA in tumor and corresponding normal noncancerous tissues. *P<0.05, **P<0.01. A. *PLCE1* mRNA expression in tumor and corresponding normal noncancerous tissues in total samples and samples stratified by different genotypes of rs2274223. B. *PLCE1* mRNA expression in tumor and corresponding normal noncancerous tissues in total samples and samples stratified by different genotypes of rs11187870. C. *PLCE1* mRNA expression of different genotypes for rs2274223 and rs11187870 in normal noncancerous tissues in tumor site of cardia and noncardia.

To identify a possible association of rs2274223 and rs11187870 with *PLCE1* mRNA levels, we further analyzed *PLCE1* expression levels of tumor and adjacent noncancerous tissues from patients with different genotypes. A total of 48 gastric cancer tissues and corresponding normal noncancerous tissues were subjected to the real-time PCR to quantify *PLCE1* transcript levels. As shown in Figure 2A, the expression levels of *PLCE1* mRNA was significantly lower in tumors than in adjacent noncancerous tissues in all samples, about 0.41 fold compared with noncancerous tissues. (0.019±0.002 vs. 0.008±0.001, P<0.05). A higher expression level of PLCE1 mRNA levels was observed in both AG and GG carriers for rs2274223 in the controls (about 1.22 fold in AG carriers and 1.09 fold in GG carriers versus AA carriers) but a lower level in the GG carriers of the cases, compared to that of the AA carriers of the cases. As shown in Figure 2B, the expression of *PLCE1* by genotypes of rs11187870 was very close to that of rs2274223, and there was no significant difference in *PLCE1* expression among the three genotypes in adjacent normal tissues.

Because the risk associated with the combined rs2274223 variant AG/GG genotypes and rs11187870 variant CG/CC genotypes was more evident in subjects with cardia cancer, we also compared the *PLCE1* expression in subtypes of cardia gastric cancer and non-cardia gastric cancer. In cardia gastric cancer, GG carriers showed a higher expression of *PLCE1*(1.08 fold, compared with that AA carriers; *P* = 0.91), while in noncardia gastric cancer, AG carriers showed a 1.52 fold higher expression of *PLCE1* than that of the AA carriers (*P* = 0.96) (Figure 2C).

## Discussion

In this case-control study, we investigated the associations of two novel, potentially functional SNPs, one located in exon 26 (rs2274223A>G) and the other located in the 3′ UTR (rs11187870G>C) of *PLCE1*, with risk of gastric cancer in an eastern Chinese population. The variant genotypes of each SNP were associated with increased risk of gastric cancer, and this risk was more evident in subgroups of males, non-smokers, non-drinkers and patients with gastric cardia adenocarcinoma. In the combined analysis, the joint effect of risk alleles contributed to the risk of gastric cancer in an allele dose-response manner. Therefore, our findings support the hypothesis that potentially functional SNPs of *PLCE1* may play a role in the etiology of gastric cancer. This finding is consistent with that reported in two recent GWASs of northern Chinese populations [13], [14] and another independent replication study [15].

The *PLCE1* gene is a member of the phospholipase C family [22], [23], [24]. The PLCE1 protein contains one CDC25 domain at its amino terminus that functions as a GEF for H-Ras and/or Rap1. Two RA domains located at the carboxyl terminus of PLCE1 are associated with H-Ras and Rap1A in a GTP-dependent manner. These structural features suggest that PLCE1 is regulated downstream of the Ras superfamily GTPases [25], [26]. PLCE1 catalyzes the hydrolysis of polyphosphoinositides into two intracellular second messengers, such as inositol-1,4,5 trisphosphate (IP_3_) and diacylglycerol (DAG) that are involved in calcium mobilization and protein kinase C activation, respectively [25], [27]. Therefore, PLCE1 acts as an effector of the Ras family small GTPases and thus plays an important role in regulating cell growth, differentiation and development. There is evidence that PLCE1 may be associated with pathogenesis; for example, positional cloning revealed that mutations in *PLCE1* were responsible for the early-onset nephrotic syndrome [28] and that the relatively frequent mutations in *PLCE1* might cause isolated diffuse mesangial sclerosis (IDMS)[29]. Recent studies also have shown that PLCE1 could play crucial roles in intestinal tumorigenesis and skin tumor formation [30], [31]. It is likely that the PLCE1 may play a role in tumorigenesis by a mechanism of augmentation of angiogenesis and inflammation responses [30], [31]. To date, only two recent GWASs [13], [14] and a replication study [15] have investigated the association between genetic variations of *PLCE1* and susceptibility to gastric cancer.

In the present study, we confirmed the reported association between the *PLCE1* rs2274223A>G SNP and gastric cancer susceptibility [13], [14], [15]. Our mini-meta analysis results showed that the risk estimates of the AG or GG genotype vs. the AA genotype in our study were similar to that of the other three published studies. In addition, the 3′ UTR rs11187870G>C SNP of *PLCE1* was identified for the first time to be associated with increased risk of gastric cancer, although this SNP is in incomplete LD with rs2274223A>G. It is likely that this SNP in the 3′UTR may disrupt the microRNA–mRNA interaction and affect expression of the microRNA targets [32], [33]. In the combined analysis, subjects who carried more risk alleles (i.e., rs2274223G and rs11187870C) of *PLCE1* showed a higher risk of gastric cancer, suggesting a joint effect of these two SNPs on gastric cancer susceptibility. In further logistic regression analysis by simultaneously adjusting for each of the two loci, only the risk associated with rs2274223 remained statistically significant, which may reflect the fact that rs11187870 is in incomplete LD with rs2274223. In the haplotype analysis, compared with the most common A_rs2274223_G_rs11187870_ haplotype, the G_rs2274223_C_rs11187870_ and G_rs2274223_G_rs11187870_ haplotypes were strongly associated with an increased risk of gastric cancer. Taken together, the potentially functional rs2274223G and rs11187870C alleles are most likely to be responsible for the observed risk associated with genetic variations in *PLCE1*.

In the stratified analysis, the combined effect of rs2274223 and rs11187870 SNPs was more evident in males, non-smokers, non-drinkers and cardia adenocarcinomas. These findings are consistent with the concept of genetic susceptibility, in which individuals at risk are likely to develop cancer when they have been exposed to a low level of exposure. There are several possibilities for such findings. First of all, by definition, “non-smokers” and “non-drinkers” were those who may have exposed to low levels of smoking or alcohol, and never smokers may have been exposed to passive smoking or other unknown carcinogens in the environment. For smokers and drinkers, the effect of genetic variations may be overwhelmed by the strong impact of environmental carcinogens. In contrast, for those who exposed to low levels of smoking or alcohol, genetic variations may play a dominant role in the initiation of carcinogenesis. Secondly, our study is still not large enough to provide enough statistical power to detect any gene-environment interaction. Therefore, larger population-based studies, preferably with detailed information about passive smoking, are required to further validate our findings. As cancer is a complex and multifactorial disease, gene-gene and gene-environment interactions may occur, and a single genetic variant is insufficient to predict the overall risk. Thus, future studies should include more functional SNPs in *PLCE1* or in other related genes in the similar biological pathways that may be involved in the etiology of gastric cancer.

To verify whether rs2274223 would affect *PLCE1* expression and thus play a role in the etiology of gastric cancer, we used the real-time PCR to measure *PLCE1* expression at the mRNA level. Overall, the mRNA expression level was much lower in the tumors than in the adjacent normal tissues, which was consistent with two separate studies on colorectal carcinoma [34], [35]. In these two studies, over-expression of *PLCE1* caused a higher rate of cell death, inhibition of proliferation or promoted apoptosis in colorectal tumor cells, indicating that *PLCE1* might negatively regulate viability and proliferation of colorectal tumor cells and thus might act as a tumor suppressor gene [34], [35]. In the present study, there was a non-significantly higher expression of *PLCE1* mRNA in AG and GG carriers than in AA carriers in the controls; however, such a difference did not show in the GG carriers of the cases. This discrepancy is either due to limited samples used for the detection or the cases with the GG genotype may have other unknown defects, such as lost of heterozygosis, because the GG genotype was associated with much lower *PLCE1* expression in the cases but not in the controls. Meanwhile, higher expression was observed in subjects carrying the AG genotype but not the GG genotype. This may be due to the relative small sample size for the real time PCR analysis or there were some unknown mechanisms, e.g., *PLCE1* may be regulated by other genes involved in cell growth and differentiation. The expression levels of *PLCE1* were also different between cardia and noncardia tumors, that is, there was a higher expression level in GG carriers in cardia cases but a higher expression in AG carriers in noncarida cases. This may also be due to the limited sample size of cardia cases. Therefore, the expression of *PLCE1* was not found to be influenced by this polymorphism in our study, suggesting that some unknown consequences of the polymorphism, rather than a quantitative change, may be associated with the development of gastric cancer. It is also possible that the expression of *PLCE1* may be linked to some other polymorphisms within *PLCE1* or elsewhere in the genome, as well as some other factors that may enhance or repress its expression. In addition, the sample size of the current study was relatively small, and larger study is needed to confirm the results of our present study and better clarify the implication of the genetic background of *PLCE1* on the development of gastric cancer.

Several limitations of our study need to be addressed. Firstly, this was a hospital-based case-control study with patients from hospitals and controls from the community, and thus selection bias cannot be completely excluded. However, potential confounding bias may be minimized by frequency-matching cases and controls on age, sex, areas of residence and further adjustment for potential confounding factors in final analyses. Second, the sample size of our study was moderate, and the statistical power of the study may be limited, particularly for the stratified analysis and detection of gene-environmental interactions, although our mini-meta analysis suggested that the risk estimate of variant genotype in our study was very close to that of other published studies. Third, only two potentially functional SNPs of *PLCE1* were genotyped in this study, which did not cover all variants of *PLCE1* and restricted further haplotype analysis. Fourth, the functional analysis in the form of mRNA expression levels may be rudimentary, further functional analysis with immunohistochemistry for rs2274223 in exon and plasmid construction for luciferase assay for rs11187870 in 3′UTR are needed to unravel the underlying mechanism. Finally, information on other exposures, such as dietary intake, occupational exposure and *Helicobacter pylori* infection, were not available for analysis. Future studies need to address whether these factors interact with genetic variants in *PLCE1* in the etiology of gastric cancer.

In conclusion, in this case-control study of gastric cancer in an eastern Chinese population, we provided statistical evidence that confirmed the associations between the reported *PLCE1* rs2274223, as well as the novel rs11187870, and risk of gastric cancer. Consistently, the combined effect of these two SNPs and their estimated haplotypes were also associated with gastric cancer risk, suggesting that genetic variations in the *PLCE1* gene may play a role in the development of gastric cancer. However, these findings call for larger and more in-depth molecular studies that are needed to unravel the role of rs2274223G and rs11187870C alleles in the etiology of gastric cancer.
